# Bridging the European Data Sharing Divide in Genomic Science

**DOI:** 10.2196/37236

**Published:** 2022-10-19

**Authors:** Fruzsina Molnár-Gábor, Michael J S Beauvais, Alexander Bernier, Maria Pilar Nicolas Jimenez, Mikel Recuero, Bartha Maria Knoppers

**Affiliations:** 1 Faculty of Law University of Heidelberg Heidelberg Germany; 2 Centre of Genomics and Policy Faculty of Medicine and Health Sciences McGill University Montreal, QC Canada; 3 Faculty of Law University of Toronto Toronto, ON Canada; 4 Research group in social and legal sciences applied to the new technosciences IT 1541-22 Faculty of Law University of the Basque Country, Basque University System Bilbao Spain

**Keywords:** international data transfer, scientific research, genomics, safe data spaces, data protection

## Abstract

In this viewpoint, we argue for the importance of creating data spaces for genomic research that are detached from contexts in which fundamental rights concerns related to surveillance measures override a purpose-specific balancing of fundamental rights. Genomic research relies on molecular and phenotypic data, on comparing findings within large data sets, on searchable metadata, and on translating research results into a clinical setting. These methods require sensitive genetic and health data to be shared across borders. International data sharing between the European Union (EU) or the European Economic Area and third countries has accordingly become a cornerstone of genomics. The EU General Data Protection Regulation contains rules that accord privileged status to data processing for research purposes to ensure that strict data protection requirements do not impede biomedical research. However, the General Data Protection Regulation rules applicable to international transfers of data accord no such preferential treatment to international data transfers made in the research context. The rules that govern the international transfer of data create considerable barriers to international data sharing because of the cost-intensive procedural and substantive compliance burdens that they impose. For certain jurisdictions and select use cases, there exist practically no lawful mechanisms to enable the international transfer of data because of concerns about the protection of fundamental rights. The proposed solutions further fail to address the need to share large data sets of local and regional cohorts across national borders to enable joint analyses. The European Health Data Space is an emerging federated, EU-wide data infrastructure that is intended to function as an infrastructure bringing together EU health data to improve patient care and enable the secondary use of health-related data for research purposes. Such infrastructure is implementing new institutions to support its functioning and is being implemented in reliance on a new enabling law, the regulation on the European Health Data Space. This innovation provides the opportunity to facilitate EU contribution to international genomic research efforts. The draft regulation for this data space provides for a concept of data infrastructure intended to enable cross-border data exchange and access, including access to genetic and health data for scientific analysis purposes. The draft regulation also provides for obligations of national actors aimed at making data widely available. This effort is laudable. However, in the absence of further, more fundamental changes to the manner in which the EU regulates the secondary use of health data, it is reasonable to believe that EU participation in international genomic research efforts will remain impeded.

## The General Data Protection Regulation Hurdle for International Genomic Research

The launch of many large-scale multinational research projects over the past 2 decades exemplifies the importance of international data sharing in genomics and omics research [1,2]. Cross-matching data between centers, establishing large community reference data collections, and accessing external reference data sets enhances the understanding of human biology and disease and benefits translational stratified medicine.

Thus, data protection issues related to international data sharing are inextricably linked to genomic research. Furthermore, where data protection issues are considered in an international context but with European Union (EU) involvement, reference to the EU General Data Protection Regulation (GDPR) [3] becomes unavoidable.

This regulation is perceived to hinder rather than promote cross-border data sharing at the international level [4]. The main objective of the GDPR is to create nuanced rules that balance the benefits and risks of processing personal data and protecting individual interests. The international data transfer mechanisms of the GDPR act to ensure that the obligations applicable according to EU data protection law continue to be applied after the data are transferred outside the EU and the European Economic Area, including the proportionate balancing of benefits and harms.

Regulators and courts often deem unlawful outbound transfers of data from the EU and European Economic Area to third countries that implement considerable State surveillance measures. Such legal determinations act as a functional bar to the outbound transfer of genomic data of EU and European Economic Area provenance to such third countries. Furthermore, such a legal determination places on prospective data transferors the considerable burden of assessing the State surveillance practices of third countries before performing international data transfers in favor thereof. This heightens the complexities and compliance costs inherent in performing outbound data transfers from the EU and the European Economic Area to third jurisdictions.

Thus, any concerns about fundamental rights arising in a third country in relation to surveillance activities carried out by public authorities influence the assessment of the level of protection in that country and lead to its rules being found disproportionate or even disregarding the core of the fundamental rights concerned. This practice precludes outbound transfers of genomic data from being performed unless significant political changes are made to the policing and surveillance practices of those third countries [5]. Although it is crucial to raise the level of data protection in relation to the surveillance activities of State actors from a fundamental rights perspective, this is a long-term endeavor. Until this is achieved, data sharing for socially significant purposes such as scientific research will decline. Removal of these considerable barriers to data sharing in genomic research is contingent on political determinations that are outside the scope of activities of scientific research communities.

In this paper, we argue for the importance of creating data spaces to enable international collaboration in the use of genomic data. These data spaces should be detached from contexts in which fundamental rights concerns related to surveillance measures override a purpose-specific balancing of fundamental rights and of the benefits and risks of processing personal data and protecting individual interests. First, we assess the relevant provisions of the GDPR and detail their implications for data exporters and importers. Second, we outline how State surveillance practices can affect the potential for researchers to share genomic data. Third, we address the fundamental rights context of scientific research. Fourth, we analyze possible solutions that would enable genomic data to be transferred to researchers in countries outside the EU and the European Economic Area without international consensus being achieved on issues of State surveillance. Contractual issues are first discussed, followed by secure data spaces. This structure allows us to address the challenges of international data transfers in the current legal situation in detail, identify the main problems, and, on this basis, consider which solutions could provide a remedy, also in the context of further clarification of the cornerstones of the European Health Data Space (EHDS).

## Background: GDPR Transfer Rules

In this section, we describe in considerable detail the international data transfer rules of the GDPR. This discussion provides the necessary context in framing the challenges that the international data transfer rules create for genomic researchers. This description is necessary to demonstrate how our proposed solution responds to the needs of scientific researchers and would also meet the demands of EU and European Economic Area data protection regulators.

The main legal mechanism by which personal data may be transferred from the EU and the European Economic Area to a third country for scientific research purposes is a decision of the European Commission confirming the adequacy of the level of data protection in the recipient country.

The European Commission is responsible for determining whether a third jurisdiction is adequate. Once a jurisdiction has been deemed adequate, outbound transfers of data to that third jurisdiction can be made without additional legal compliance efforts being required. If the destination of an international data transfer is not subject to an adequacy decision, additional measures enabling legal compliance must be implemented before the outbound transfer is performed.

The following criterion is used to determine whether a third jurisdiction can be deemed *adequate*. An *adequate level of data protection* requires that the third country ensure, by virtue of its domestic legislation and international commitments, a level of data protection “essentially equivalent” to that guaranteed in the EU [6].

This does not mean that an identical level of protection is required. The methods used to protect data by the third country may differ from those used in the EU, but such methods must nevertheless prove *effective in practice* [7]. The GDPR also defines rules guiding the assessment of adequacy as to whether the essence of the fundamental right to data protection is respected and whether its limitation is subject to the principles of necessity and proportionality.

The text of the applicable laws in the concerned jurisdiction is not the sole criterion assessed in performing this evaluation. Indeed, the practices of authorities, administrative bodies, and courts in the country of destination are of equal relevance in assessing whether adequacy status can be lawfully conferred. Decisions of the European Commission establishing private sector areas in the United States as adequate for the purpose of receiving data transfers from the EU have been annulled twice by the Court of Justice of the EU (CJEU). This court is the principal court that is responsible for interpreting the fundamental right to data protection in the EU [5,6]. The annulment of these decisions was rooted in concerns about the fundamental rights to data protection, respect for private life, and effective remedies (ie, the availability of redress mechanisms for affected parties) as defined by the EU Charter of Fundamental Rights, a legally binding catalog of human rights applicable in the EU (cf Article 7: Respect for private and family life, Article 8: Protection of personal data, and Article 47: Right to an effective remedy and to a fair trial). In this decision, the CJEU concluded that the surveillance practices of US authorities and the lack of redress available to EU citizens relative thereto violated the aforementioned human rights guarantees [8].

Therefore, in summary, the European Commission is responsible for ascribing *adequacy* status to countries outside the EU and the European Economic Area. This requires the European Commission to determine that the concerned jurisdiction provides data protection guarantees *essentially equivalent* to those available in the EU.

Recent adequacy decisions portend a change from a relatively lenient adequacy analysis to a more stringent evaluation that requires the legislation and administrative practices in the concerned jurisdiction to mirror those in the EU. This is seen very clearly in the case of Japan [9]. In Japan, the adequacy decision of the European Commission related only to the private sector, considering that oversight mechanisms in data protection law differ in their design in the private and public sectors. Such restrictions on the scope of adequacy can be understood as a strong indicator that sector-specific evaluations of foreign data protection legislation will, in the future, be used to confer adequacy status on a sector-specific or statute-specific basis rather than on a national basis. Given the rigor and granularity of recent European Commission adequacy analyses, it is not surprising that the level of data protection is currently confirmed by an adequacy decision of the European Commission in only 13 countries and territories around the world [10].

## Implications of the GDPR Transfer Mechanisms for Data Importers and Exporters

If data are transferred from the EU or the European Economic Area to a jurisdiction that is not subject to an adequacy decision, a distinct legal mechanism must be implemented to ensure the lawfulness of the data transfer. The level of data protection in the third country must be examined not only in the case of an adequacy decision but also when the international data transfer is based on another transfer mechanism established in the GDPR [5]. The categories of available transfer mechanisms include the imposition of additional safeguards that maintain the standard of data protection in the EU and the European Economic Area, which are exhaustively enumerated in the text of the GDPR. At the time of writing, the only transfer safeguard that has been developed in a standardized manner are the Standard Contractual Clauses. These Standard Contractual Clauses require transferors to integrate standard-form contractual language into their data transfer agreements, which imposes numerous GDPR-derived compliance-related obligations on the recipients of the data transfer, effectively approximating the extraterritorial application of the GDPR. The other transfer mechanism is a context-specific derogation from the application of the GDPR to enable a specific transfer that could not otherwise be performed in compliance with the requirements of the GDPR. There is a considerable range of derogations that are potentially relevant to international transfers of genomic data. However, these derogations are intended to enable personal data to be transferred out of the EU or the European Economic Area on an ad hoc, exceptional basis. Therefore, Standard Contractual Clauses are the only real mechanism that could at present enable continuous, ongoing international transfers of personal data to jurisdictions that do not benefit from an adequacy decision.

Senders and recipients of personal data who base their data exchange on contractual clauses such as the Standard Contractual Clauses of the European Commission are also obliged to verify before each transfer whether the level of data protection secured under EU law is met in the recipient country. Accordingly, the Standard Contractual Clauses can only be drawn on to secure international data transfers if the national legislation in the country of data import allows the data recipient researcher to comply with the contractual provisions [11].

However, it is unclear whether this can realistically succeed. Researchers cannot bind themselves to rules contradicting their obligations under domestic law, such as requirements to disclose data to local authorities. Furthermore, in some countries—such as the United States—researchers are often subject to legislation that prevents them from signing the Standard Contractual Clauses. In addition, their potential to enhance data subjects’ protection by, for instance, establishing institutional complaint mechanisms has only a limited effect on the actual improvement of the fundamental rights protection of those affected if administrative and judicial remedies in the concerned jurisdiction cannot safeguard the fundamental rights of EU citizens. Hence, even the recent call for special contractual clauses for the scientific research processing sector [12] falls flat if the regulatory environment does not secure a comparable level of fundamental rights protection for the data subject equivalent to that of the GDPR. In this case, the concerned sector cannot be deemed adequate as other applicable rules (related to compliance with national surveillance mechanisms) preclude researchers from contractually binding themselves to terms that approximate those of the GDPR. As a consequence, research endeavors involving a transfer of personal data from the EU or the European Economic Area to the United States often cannot be implemented in many cases, such as sending deidentified human genetic data to the Imputation Server hosted by the University of Michigan or pooling personal data on a single server as envisaged by the International Alzheimer’s Consortium and the US-based Alzheimer’s Disease Sequencing Project [13].

Performing an assessment of the law and practice of the jurisdiction of a destination is referred to in the literature as a “mini-adequacy decision” [14]. With each transfer, data exporters are required to consider the ease of access to data by government actors, the possibility for the simplified exercise of rights and effective remedies for breach thereof, and whether the destination of the transfer is governed by the “rule of law.” Performing such an analysis in individual cases and monitoring changes in local law and government practices is likely to exceed the capabilities of researchers exporting data, who are obligated to perform the test in the first place. There is still no helpful guidance on how data controllers responsible for defining the purposes and essential means of data processing are supposed to perform a task that the European Commission has failed to tackle on more than one occasion, as evidenced by the annulment of its adequacy decisions by the CJEU. Shifting the burden of an all-encompassing assessment of a third country’s legal system to exporters of data might lead to quasi-arbitrary evaluations as well as to divergences in the application of the adequacy criteria from one another [15]. If these evaluative exercises are carried out poorly, it could lead to the erosion of the fundamental rights of the affected EU citizens. This could take a long time to remedy as the CJEU is the only body competent to do so, and it requires more than a year to decide cases [16].

## Do Anonymization or Security Measures Offer a Solution?

According to the European Data Protection Board, the group of national supervisory authorities interpreting the GDPR, any anonymization must be completely irreversible. Some further consider that anonymization must be *future-proof* such that anonymization is impervious to new technologies not yet invented. Indeed, the European Data Protection Board presents the deletion of original data and the removal of characteristics as ideal technical measures of anonymization [17,18].

An exigent threshold for what constitutes anonymized data is a barrier to international genomic research. In health-related genomic data processing, this approach poses acute difficulties as the interrogation of disease etiology and other determinants of health requires personal data. Deleting or stripping data sets of certain variables in the name of anonymization is then directly opposed to the very reason for which processing is undertaken. Examples include cancer imaging data—the anonymization of head and neck images constitutes a serious challenge to the preservation of essential scientific data; as such, modification of the data can diminish their scientific quality and utility [19]. In addition, removing metadata of a specific format (eg, Digital Imaging and Communications in Medicine) [20] from cancer imaging data sets that may constitute indirect identifiers (eg, the manufacturer’s serial number) would imply the loss of traceability of the patient. A loss of traceability can have particularly severe consequences in international clinical trials where the ability to follow patients is essential and the identifiable verification of the study results constitutes a legal duty in many countries; their return to the patients must represent an inherent part of the study concept [21]. Furthermore, data anonymization methodologies can systematically deprive the members of small population groups, including traditionally marginalized groups, from inclusion in scientific data sets. This is the case as the indirect identifiers of small population groups are less common than those of majority groups and, therefore, deidentification methods tend to remove them from data sets more often [22].

As a result, the advantages of anonymization cannot be realized in a research context without drastically reducing the potential of the research. Decreasing the richness of data diminishes their scientific value, further limiting the research questions that the data can address, the applicable research methods, and the relevance of the research findings. In addition, divergent data quality will ultimately reduce interoperability between data sets and may even affect the reproducibility and comparability of research results, destroying their statistical validity [23].

Methods other than data anonymization, such as coding and encryption, cannot necessarily facilitate the international transfer of personal data. These methods do not preclude the application of the GDPR to the data. The European Data Protection Board considers coding and encryption methods to be supplementary measures enhancing data protection compliance efforts rather than anonymization techniques that render data nonpersonal.

It seems that security measures widely used in genomic research, such as pseudonymization, cannot remedy this issue, either. The European Data Protection Board considers encryption methods and coding, such as pseudonymization, to be supplementary measures enhancing data protection rather than measures that render data nonpersonal and, thus, outside of the material scope of the GDPR [24]. Accordingly, GDPR requirements for transfer cannot be fulfilled in most cases if it is not possible to protect encrypted and coded data against large-scale access and monitoring by the third countries’ law enforcement agencies without corresponding administrative and judicial remedies.

## Consequences of the Current Rules

In summary, it can be stated that all mechanisms offered by the GDPR to secure admissibility of international data transfers can only be applied if the recipient outside the EU or the European Economic Area provides an *essentially equivalent* level of data protection. The fundamental rights context surrounding data processing in the recipient country will influence the assessment of whether rules defining data processing in a certain sector are essentially equivalent to the GDPR standard. This means that the burden of investigating adequacy in recipient countries without an adequacy decision by the European Commission will ultimately lie with the data exporter. At the same time, data recipient researchers will need to determine whether they can sign the offered GDPR Standard Contractual Clauses and adhere to them or whether there exist contradicting obligations for them based on national laws. These rules place a burden on researchers in an era where compiling large data sets across cohorts and countries is crucial for achievements in genomic science.

What happens currently to a data transfer to a third country without an adequate level of data protection? In the absence of an adequacy decision and in the event that Standard Contractual Clauses or other transfer mechanisms cannot ensure an essentially equivalent level of data protection in the country of the data recipient, the controller must provide additional security measures that effectively prevent external access to the data and, thus, protect the rights of data subjects. The function of additional measures is similar to that of any transfer mechanism: they should *compensate* for the lack of high-level data protection that is essentially equivalent to that in the EU and the European Economic Area. These additional measures include the anonymization of all personal data or privacy-preserving techniques such as encryption and coding, where only the data exporter has the key and which cannot be circumvented by others [24].

Anonymization is not a viable solution to circumvent the application of data protection rules. On the one hand, genomic data are highly identifiable because of further data linkages. In contrast, the benefits of genomic research in a health context can only be achieved regularly if there is at least a stratified possibility of tracing the data back to the affected patients and probands.

Concerning privacy-preserving techniques, a discrepancy between technical measures and the standard of “essential equivalence” emerges. Adequacy is fundamentally a legal standard that includes considerations of data access by authorities and the legal obligation of data importers to comply with access orders. Depending on the legal safeguards and available redress mechanisms, the order and corresponding obligation to comply with it may *in themselves* create a processing context for data importation that is below the standard of the EU. Furthermore, issues such as encryption and the availability of other data to bypass the contextual anonymity offered by pseudonymization (coding) are technical. Overturning technical data security will allow for the application of a processing context that would render protection inadequate. Although researchers will only be able to influence technical measures applied to their data processing, the lawful access by the recipient countries’ authorities and its interrelatedness with the technical factor needs to be dealt with.

## Emerging Solutions to Lawful Data Access by Third-Country Authorities

Generally, the task of assessing whether the level of data protection in a third country is equivalent in substance to the level under EU law is not a mechanical exercise but must involve sophisticated analysis of the legal order of the third country. The analysis must not only cover all areas of law in terms of legislation and case law but also further extend to administrative practices. That is, a study of the literal text of the law is insufficient. Facts on the ground, such as actions taken by administrative bodies, also matter. Evaluating the conservatory measures that the recipients of international data transfers take against orders or requests for information from law enforcement agencies and surveillance bodies could inform the assessment of the “essential equivalence” of a recipient jurisdiction’s legal system and afferent practices.

In Canada, for example, some entities make it clear that they will only comply with a valid court order from law enforcement agencies. When reading the transparency reports of these organizations, it becomes clear that most requests are not legally authorized as law enforcement agencies are essentially “asking” for access but cannot compel it [25]. Even if there is a court order, it might only cover limited data sets independent of the collective access for which a law enforcement agency has asked. Canadian human-participant research norms, which are binding on federally funded research, underscore the obligation of both researchers and their institutions to uphold promises of participant confidentiality, which can require researchers and research institutions to contest court orders for data [26].

Although there is little that can be done about surreptitious surveillance, procedures requiring data recipients to contest authorities’ requests and orders for access to personal data should be considered when determining whether an international transfer respects the fundamental rights of data subjects. We believe that the analysis of administrative practices that protect fundamental rights will reveal considerable similarities between EU and non-EU legal systems, which are more telling than the superficial differences arising from the formal comparison of the texts of EU and non-EU data protection legislation.

Countries to which the European Commission has denied GDPR adequacy status or that have had their adequacy status overturned by decisions of the CJEU have taken action to heighten the protection of fundamental rights that is accorded to their citizens. In the United States, data protection rights such as the “right to deletion” are now acknowledged in case law [27]. In addition, some countries that did not offer administrative and judicial data protection remedies to foreign citizens are starting to do so [28]. In Japan, independent administrative oversight has hitherto only been acknowledged in the context of private sector use [29]. Soon, a novel adequacy decision applicable to personal data processing by public sector researchers might be implemented in Japan, extending protection with regard to the public sector use of data. Altogether, significant progress will still be required to raise fundamental rights protection in relation to public authority surveillance to a globally standardized level. Data sharing for scientific research and health care purposes is a pressing global health concern and a predicate for achieving health equity. The pursuit thereof cannot be made contingent on global consensus on issues of State surveillance. Precluding the international exchange of genomic and health data for political reasons condemns at-risk populations to bear poor health outcomes to place pressure on governments to align on surveillance policy.

## Scientific Research: Its Technique and Legal Status

Once within the scope of the application of sector-specific data protection law, it is primarily the context of genomic research that will guide the—often complicated—weighing exercise of competing rights and interests, such as between research freedom and data protection, both of which are fundamental rights capable of being limited. In addition, other legally relevant positions on the side of the data subject, such as their right to health and their right to decide what to do with their data (right to private and family life), may move the metaphorical scale in favor of genomic science in certain contexts. In genomics, affected patients may have significant, real opportunities to benefit from research findings, for instance, the clinical validity of a genetic mutation or when a variant is confirmed through translational scientific research.

It is an outstanding achievement as to how far this weighing has been enhanced on the legislative level in the GDPR, where the emphasis on research freedom is strongly guided by the relevance of scientific endeavors in the public interest and permits the data subjects’ data protection interests to be limited while at the same time striving to minimize risks for their privacy [30]. Under the GDPR specific regime for scientific research, the primary role of data security is to mirror the outcome of the trade-off between the main interests of processing intrinsic to scientific freedom and those of privacy in the context of research, with other important interests such as those of the public guiding the trade-off. Obligations for data controllers and processors to implement technical data security measures as defined by the GDPR generally and for scientific data processing specifically (eg, Article 89 safeguards that mandate data minimization and pseudonymization) address the trade-off result between these very interests and rights at the legislative level. Altogether, supplementary measures completing data protection in the genomic data governance context, particularly technical security and administrative protections against enforced data release, and the oversight of future use introduce a cumulative practice of good governance rather than a “silver bullet” consisting of a singular method that alone guarantees data protection, as is implicit in the discussion of secure multiparty computation and other technological measures in recent European guidance [24].

## Scientific Research as an Element of Legal Weighing Exercises

Weighing competing rights and interests and translating the result of this balancing exercise into practices, policies, and technical measures enabling secure genomic data exchange will become more complex in the future. Historically, the analysis has required the bilateral consideration of the individual interest in the protection of patients and research participants relative to the research freedom of scientists and the broad societal interest in advancing research and delivering a high standard of health care. However, the relevant interests are now becoming multipolar. Namely, the balancing of interests to be performed becomes multipolar. It becomes necessary to consider not only the privacy interests of individuals relative to scientific freedom and the public interest in scientific progress but also the additional complexities of State surveillance directed at individuals and the public.

When the adequacy of a third country’s data protection is contested on the basis of law enforcement’s potential access to data and the removal of data anonymity, the initial weighing context related to scientific research and the GDPR’s privileging of data processing for scientific research purposes shifts considerably.

The essentially bilateral relationship between privacy and scientific freedom until then determined the appropriate security measures to be implemented. However, where there is a prospect of surreptitious State surveillance or of law enforcement access to data, the security measures and other safeguards to be implemented differ. The choice of appropriate security measures and other safeguards must account for the potential for State surveillance or law enforcement access to data, which is a different analysis altogether. In these circumstances, the assumption of contextual anonymity that undergirds the governance of data for scientific purposes might be more easily dissolved than assumed, especially in relation to the identifiability of genomic data and with the technological tools available to law enforcement [31].

Furthermore, it seems misleading to frame data protection obligations within the binary distinction between anonymized data and personal (including pseudonymized) data. As already described, the anonymized-personal binary is not a pertinent distinction in the context of the decision as to whether processing data for scientific research purposes and transferring them to third countries can occur in a manner compliant with the GDPR. It is personal data to which the rules of the GDPR apply, including rules on transfer. The risk of identifiability is thus implied in the data security and transfer mechanisms of the data protection law and the rules of the GDPR.

The issue here is that the main technical measures that would still enable meaningful and beneficial genomic science are contradicted by considerations surrounding the legal and de facto possibility of law enforcement circumventing contextual anonymity. We believe that there needs to be a distinct legal framework enabling scientific research as the currently proposed solutions pose challenges to researchers that they cannot solve on their own (Table 1). This framework should protect, where necessary, scientific research from other interests. The creation of such a framework should be based on a normative decision instead of its facilitation being only dependent on the technical agility of data exporters and importers. The main concept of the proposed legal framework is elaborated on in the following sections. Significant elements of the concept are the further development of contractual obligations for data importers, creating safe data spaces, and working toward linking these with data infrastructures worldwide.

**Table 1:** Identified challenges related to international data transfers, solutions currently explored, remaining challenges, and proposed solutions to these challenges.

Challenges	Explored solutions	Remaining challenges	Suggested solutions
Lack of adequacy decision by the European Commission	Creation of clear rules for the adequacy assessment procedure	Blanket adequacy decisions easily disregard sectoral differences in applicable data protection rules	Adopt specific health research sector adequacy assessments to take into account specific trade-offs among rights, appropriate technical measures, and long-standing compliance efforts within the sector, including administrative measures
Data anonymization and privacy-preserving data security measures are promoted as the only solutions to data protection concerns	Nonapplication of data protection rules and instead use substitute measures to meet the adequacy standard	Loss of information content of data for scientific research, anonymity of data is context dependent, and substitute measures for protection can be circumvented	Emphasize the contextual anonymity of data, for example, when the context is not changed during data processing (eg, by allowing data to be visited)
Missing codes of conduct and certification mechanisms	Bottom-up sector-specific concretization of data protection rules and appropriate supplementary security measures	Current solutions that are not relevant to the context of genomic research or are not relevant for international data transfers [32]; fundamental rights issues raised	Link the development of codes of conduct with the sectoral adequacy assessment and the development of certification mechanisms with supplementary technical measures for international data transfers

## The Way Forward I: Adapting Contractual Settings

To perform a data transfer from the EU or the European Economic Area to a third jurisdiction that does not meet the “European essential guarantees,” it is necessary to apply effective supplementary measures that raise the standard of data protection to that enshrined in the GDPR. These measures can include a combination of technical measures, private law arrangements, and organizational practices.

In the absence of effective supplementary measures, supervisory authorities will bear the obligation of determining whether contractual and organizational best efforts to mitigate the potential for surveillance bodies and law enforcement agencies to make surreptitious use of data should be considered sufficient to enable the international transfer of data [31]. Potentially relevant measures include the integration of contractual language mandating mutual transparency to agreements between data importers and exporters. This might include the obligation to regularly provide specific information about requests received from authorities regarding personal data processed under the relevant contract. If disclosing specific details about such requests is otherwise prohibited, general information could still be provided (eg, warrant canaries) [33].

Moreover, the inclusion of obligations specific to the data importer merits consideration. These could include an obligation to take legal action to challenge an order to disclose personal data until all pathways to do so have been extinguished. Precedents for such measures exist in Canadian research ethics guidance [26]. These recommendations are often paired with suggestions for creating joint liability between the data exporter and the recipient as well as with rules for compensation, such as the inclusion of an obligation for the data importer to indemnify the data subject, regardless of fault, against all damage caused by access to the data subject’s data by entities of their State. Issues related to the effective enforcement of such additional clauses remain open. Data exporters can demonstrate and document their intention to act in a legally compliant manner by observing the requirement of the supervisory authorities and contacting the particular data importer to arrange for these changes to be made to the provisions of the contractual clauses. The stepwise escalation of discretionary measures by supervisory authorities may eventually reduce the exposure of data exporters acting in good faith to high penalties. However, it does not per se lead to an improvement in the position of data subjects as the demonstration and documentation of the will to comply with the law by the contractual parties does not necessarily guarantee enforceable rights for data subjects.

Furthermore, the proportionality of such extensions to contractual agreements will often depend on whether the data importer is replaceable in the short and medium term by an importer who may more easily guarantee an adequate level of data protection. However, this assessment standard is a double-edged sword. Although the irreplaceability of a data importer might be a good yardstick for further enabling standards in the economic sector, the irreplaceability of a scientific cooperation partner must always be judged against the backdrop of the high standards to which the exercise of the fundamental right to scientific freedom is linked. The risk remains that the replaceability of different partners in genomic science might quickly be based on a superficial comparison of available technical equipment or external indicators of success. Although these metrics can influence the exercise of scientific freedom, they must not influence the protective value assigned to scientific freedom as a *right of freedom*.

## The Way Forward II: Safe Data Spaces for Scientific Research

With the European Data Strategy, the EU aims to create a single space for data that will allow them to flow freely within the EU and across sectors for the benefit of businesses, researchers, and public administrations. One of the core pillars of this strategy is precisely the promotion of “Common European data spaces in strategic sectors and domains of public interest,” including the European Health and European Research Data Spaces [23]. The EHDS aims to enable an efficient exchange of and direct access to different health-related data across the EU in compliance with data protection regulations, in particular the GDPR [34]. As for the regulatory subject matter, the design of the rules for data exchange as well as connecting the EHDS with the emerging genomic research infrastructure is of particular relevance. The “1+ Million Genomes” is an initiative of individual EU member states that aims to enable the sharing of at least one million genomes by 2022 [35]. This initiative is particularly important as the EHDS expressly includes genomic data in its scope and should be connected to the “1+ Million Genomes” initiative in this regard.

A series of measures are proposed to foster these spaces, including the deployment of data infrastructures, tools, and computing capacity by way of scaling and interoperating repositories and databases in a federated manner [36].

Concentrating secure data processing in a cloistered data space might alleviate the imperfect regulatory environment applicable to research data processing. However, the long arm of the law enforcement regulations of third countries creates difficulties in securing the fundamental right to data protection throughout the entire life cycle of research data processing. Therefore, additional settings may be needed to help maintain data processing within a safe environment, such as preventing the download of data as a technical safeguard accompanied by the legal safeguard of contractually prohibiting it. A further step toward upholding a safe environment for research data processing is to offer a searchable metadata basis without moving data, deploying data analysis services that allow for the submission of research questions, and completely foregoing access to the research data themselves. Federated data sharing models that could ground such development are being successfully implemented by international research data archives such as the European Genome-Phenome Archive [37] and by consortia such as the European-Canadian Cancer Network [38].

In addition to processing data for primary health care purposes, the establishment of the EHDS for secondary data processing is linked to making electronic health data, health-related data already stored by various data holders, and data whose influence on health is known, such as genomic data, widely available for the purposes of health research to various data users [39]. Such data holders include public and private research institutions [40]. The draft EHDS regulation obliges data holders to make the categories of electronic data listed in the regulation available for secondary use [41]. The term “making available” means making the data available to a so-called Health Data Access Body at its request [42]. In addition, data holders are obliged to provide the Health Data Access Body with a general description of the data sets they store [43].

## Developments by the EHDS for Secondary Use of Genomic Data for Scientific Research

EU member states are required to appoint or establish public bodies entitled Health Data Access Bodies [44]. Health Data Access Bodies receive and review data users’ requests for access to data that are retained in the EHDS for secondary use, including scientific research use [45]. Prospective data users must submit requests to Health Data Access Bodies, which decide whether to authorize access to the requested data [46]. In administering such requests, Health Data Access Bodies assess a number of factors stipulated in the legislation. Relevant considerations include whether the applicant intends to use the requested data for a purpose that the law authorizes, whether the legislative preconditions to data access have been fulfilled, and whether access to the requested data is necessary for the applicant to fulfill their stated purpose [47]. If an applicant fulfills the preconditions of data access, the Health Data Access Bodies must issue a data permit in favor thereof. The permit explicitly establishes the conditions according to which the data can be used. Such a permit is valid for a maximum of 5 years [48].

The requested data are provided in either pseudonymized or anonymized form. Insofar as it is possible for the recipient to achieve their purposes in reliance on anonymized data, the data will be made available to them in an anonymized form. In all cases, data users are strictly prohibited from reidentifying the data that are provided to them [49,50].

Data access is provided through a secure data processing environment. This secure environment is subject to legislatively established security and interoperability requirements. This environment implements the technical and organizational measures required by the GDPR. For example, data users are prevented, through technical controls, from downloading data that are held in the secure processing environment [51]. The proposed legislation does not consider Health Data Access Bodies to be mere stewards of the data that are made available to data users. Rather, both the data user and the Health Data Access Body share legal responsibility for ensuring the lawful use of the requested data—the law considers them to be “joint controllers” [52].

The European Commission will collaborate with member states to create a central infrastructure that enables data users to access cross-border data through national points of contact. Member states can appoint their coordinating Health Data Access Bodies as their respective national points of contact. These contact points will become the authorized participants in the infrastructure [53,54]. National points of contact in each EU member state will compile and publish a holistic, EU-wide catalog of available data sets. This will assist prospective data users in discovering relevant data sets that are held in other EU member states for the purpose of requesting access thereto [55].

## Opening Up the EHDS for International Scientific Collaborations

The European Commission intends to enable third countries and international organizations to integrate their own national points of contact with the EHDS infrastructure. The Commission, together with the representatives of the national points of contact of EU member states, referred to as the “joint controllership group,” must perform a compliance assessment before admitting foreign nodes to the overall EHDS network [56]. If the outcome is favorable, the European Commission will adopt an implementing act, which states that the concerned foreign node is compliant with the EHDS regulation and further requirements for the secondary use of data and provides access to data users located in the EU to the electronic health data it has access to on equivalent terms and conditions [57]. Thereupon, the foreign node is admitted to the EHDS infrastructure and joins the national nodes of EU member states.

The proposed EHDS legislation establishes specialized rules applicable to the secondary processing of health data for scientific research purposes. These are compatible with the more general GDPR rules that require data protection interests to be balanced against the research interests pursued. To this end, the GDPR requires the necessity and proportionality of the intended data processing to be assessed and considered relative to the sector-specific objectives thereof.

In admitting non-EU infrastructure nodes to the pan-EU network of national points of contact, the European Commission submits the applicant foreign nodes to the aforementioned assessment procedure. By our reading, this assessment procedure mirrors the “adequacy” review that the European Commission undertakes before establishing that data importers in a third jurisdiction are authorized to receive personal data transfers from the EU without such transfers requiring additional legal compliance measures. Therefore, before foreign nodes are integrated into the EHDS infrastructure, it will be necessary for the applicant nodes from third countries to demonstrate compliance with the overall requirements of the GDPR, the EHDS regulation, and the EU fundamental rights framework to the satisfaction of the joint controllership group. This is contingent on a thorough assessment of the legal rules and practices in the applicant’s jurisdiction as regards State surveillance, among other factors.

The proposed regulation creates a relationship of joint controllership between EU national points of contact and their non-EU corollaries. This enables data subjects in the EU to assert legal claims against their own respective national EU points of contact for misuses of data that occur through the fault of non-EU points of contact. This may lead to positive outcomes in facilitating access to legal remedies for EU data subjects. However, EU points of contact could be held liable for the activities of their non-EU partners through no fault of their own, including through the breach of EU fundamental rights that arise because of the surveillance activities of non-EU State actors. This prospective liability could have a chilling effect on the joint controllership group that is responsible for determining whether foreign nodes should be admitted. That is, national EU nodes might hesitate to admit foreign nodes to the larger network if the behavior of the foreign nodes could cause the national EU nodes to be held liable for a breach of the GDPR or the EHDS regulation.

The use of a compliance assessment that mirrors the GDPR adequacy procedure to admit foreign nodes to the network of national points of contact of the EHDS is a curious legal design choice. The EHDS technical platform is anticipated to integrate secure data processing capabilities that preclude data from being externally downloaded or otherwise replicated. Regardless of the legal data protection norms—and surveillance practices—applicable in the country of origin of the contributed data, the technical design of the EHDS should achieve a common, GDPR-compliant standard of data protection guarantees. Therefore, it should be further examined whether the policy choice to require a comprehensive compatibility assessment, akin to a GDPR adequacy determination, before integrating foreign nodes into the EU network is justified at all.

The integration of national health data spaces into a larger international network will require governments and regulators to pioneer novel legislative and nonlegislative measures. In this respect, the European Commission holds a rarefied role as both lawmaker and pioneer of critical international infrastructure [12]. The European Commission has previously been criticized for not considering existing measures that are used to balance the risks and benefits of scientific research in performing adequacy assessments directed at the health sector. Perceived ambiguities arise in the guidelines of the European Commission as to the criteria that must be used to determine whether the norms of third countries should benefit from a favorable adequacy decision. This creates legal uncertainties regarding the functioning of the adequacy regime, which is the central mechanism that enables third countries to benefit from unencumbered transfers of data from the EU [58]. It remains to be seen whether the European Commission will implement transparent, comprehensible, and internally consistent methodologies in deciding on the accession of third countries’ infrastructures to the EHDS.

The GDPR continues to apply to data processing in the EHDS. Therefore, it remains open to member states to implement supplementary conditions that are applicable to data processing and international data transfer in the EHDS. The potential for member states to do so is bounded by the limits established in the GDPR. Nonetheless, this could detract from the harmonizing prospects of the EHDS in enabling distinct member states to apply their own divergent national norms to their respective nodes of the infrastructure. For example, member states can use domestic law to expressly establish limits to the transfer of specific categories of personal data to a third country or international organization for important reasons of public interest. Such limits may be imposed so long as the concerned country or international organization does not already benefit from a GDPR adequacy decision [59].

In summary, the legislation creating the EHDS reprises numerous restrictive and limitative elements of the GDPR that will continue to impede the potential to make plentiful use of data for genomic research supporting health research and care. In this respect, the EHDS will likely replicate, not resolve, the problems that the GDPR has created for international biomedical and genomic data exchange (Textbox 1). However, a foundational pillar thereof has been unduly neglected: the seamless integration of international data spaces into the EU infrastructure and convenient access to the data in the EHDS by researchers worldwide.

**Improving the European Health Data Space (EHDS).**

**Ways of improving the EHDS**

- Interpret its data sharing rules against the backdrop of necessity and proportionality of data processing for scientific research purposes
- Create detailed rules for international joint controllers assigning clear obligations to best secure the data protection rights of patients and participants
- Relieve the burden of the main rule of data anonymization for scientific data processing not to affect the quality and usefulness of research results
- Improve security and organization through further measures such as implementing data analysis services
- Reduce member states’ individual rules for data sharing through sectoral harmonization by means of certification mechanisms and codes of conduct
- Acknowledge making data available through the EHDS as a data processing step that does not constitute an international data transfer
- Acknowledge its security and organization as data sharing that is adequate for the genomic sector
- Foster public interest in genomic science through participation, information, and transparency

## Other as-yet Unused Policy Instruments to Support International Data Sharing

Having addressed how lacunae in the present draft of the EHDS legislation could inhibit equitable collaboration in international research, we now consider prospective alternatives to the current design.

The mandate to create searchable, nonpersonal data catalogs is a positive development that will help make data findable for scientific research across regions and countries. However, the EHDS legislation goes on to establish that primary data access—rather than simple data discovery—will also require the accessed data to be anonymized if identifiable data are not strictly necessary for the intended purposes. Performing scientific research using anonymized data inhibits the prospect of gaining knowledge through the analysis thereof and inhibits generalizable conclusions from being derived therefrom that can be applied to patient care.

Considering that the EHDS intends to restrict data processing to a cloistered technical infrastructure that does not enable users to download or otherwise duplicate the concerned data, the additional presumption in favor of data anonymization appears overzealous. It pursues duplicative privacy controls at little anticipated gain for data subjects while deprecating the anticipated discoveries that scientific research communities can derive through the analysis of data. At the same time, it is not comprehensible why no distinction is made within the EHDS between the assessment of the data protection standard for international scientific collaborations based on the processing of anonymous data that do not fall within the scope of data protection laws and deidentified or pseudonymized data that do.

It is recommended that the access of researchers in non-EU countries to the EHDS not be treated as an international data transfer for the purposes of the GDPR. The GDPR applies additional rules to international transfers of personal data that are directed at non-EU jurisdictions (or, rather, jurisdictions outside the European Economic Area). These rules are implemented to ensure that the standard of data protection—and the fundamental rights guarantees—that is ensured to EU data subjects is not compromised through the transfer of such data to different countries that incorporate different—and potentially lower—thresholds of data protection to their own national norms. As access to data in the EHDS is performed on EU infrastructure according to technical specifications determined by EU policy makers, it is appropriate to avoid treating such data processing activities as outbound data transfers from the EU. Indeed, there is no prospect for such data processing activities to inhibit the data protection guarantees provided to EU data subjects as both EU and non-EU access to data hosted in the EHDS take place according to the same conditions.

This determination is consistent with the CJEU’s jurisprudence, the highest court of the EU. As early as 2003, the CJEU stated that it could not be presumed that the expression “transfer [of data] to a third country” intended to include the loading of data onto an internet page even if those data were thereby made accessible to persons in third countries. If this provision were interpreted to mean that there is “transfer [of data] to a third country” every time that personal data are loaded onto an internet page, that transfer would necessarily be a transfer to all the third countries where there are the technical means needed to access the internet. This special regime would thus necessarily become a regime of general application with regard to operations on the internet [60].

The European Data Protection Board has since issued guidance that seems to contradict the foregoing case law. The Board states that an international data transfer includes not only the outright transmission of data to third parties but also acts that *make the data available* to different actors or entities in third countries regardless of whether such importers are subject to the GDPR with respect to the concerned processing activities [61].

It is uncontentious that accessing genomic data stored on an EU platform via the internet is considered a data processing operation. However, further clarification is required as to whether mere non-EU access to EU-hosted data constitutes an international data transfer. That is, the GDPR international data transfer rules are intended to be drawn on in cases where the application of non-EU data protection rules and government practices to EU-derived data has the potential to erode the privacy and data protection guarantees to which data subjects in the EU are otherwise entitled. If the EHDS data platform creates a safe data space through technical measures and data visitation requirements that ensure the continued application of EU data protection standards, it stands to reason that data processing performed on such a secure platform would not trigger the application of GDPR data transfer rules.

Regulated entities can adopt specialized tools to tailor the application and interpretation of the GDPR to a particular economic sector or sphere of activities. These include codes of conduct and certification mechanisms, among other similar tools. Implementing these mechanisms in the context of genomic research could help facilitate the outbound transfer of such data from the EU. Indeed, this is the case as the GDPR recognizes compliance with codes of conduct and certification mechanisms that the European Commission has approved as methods of ensuring the lawful outbound transfer of data from the EU to non-EU jurisdictions even in the absence of an adequacy decision in favor of the country of destination [62]. However, as with all transfer instruments intended to compensate for a lack of adequacy, the rules of such codes of conduct or certification mechanisms must be observed through binding and enforceable commitments on the part of the data recipient in the third country. These must bind the data recipients to the conditions established in the code of conduct or certification mechanism and must further guarantee respect for the fundamental rights of EU data subjects. These mechanisms bear the same limitations as other GDPR transfer mechanisms regarding the fundamental rights of EU data subjects. That is, none can overcome State surveillance practices and discrepancies in local law that would enable State actors to access the data of EU data subjects despite binding and enforceable commitments not to share such data entered into by the data recipient.

Therefore, both codes of conduct and certification mechanisms can suffer from the same imperfect fundamental rights environment as any other GDPR transfer mechanism. Despite these limitations, the aforementioned transfer mechanisms are always created in a sector-specific manner that helps specify the application of data protection rules to the particularities of the concerned data processing activities. This helps identify the technical data protection measures that are relevant to the processing activities of the concerned economic sector and balance data protection interests against other competing interests in a context-sensitive and sector-relevant manner.

## Conclusions

Providing sector-specific, purpose-related rules through codes of conduct and clarifying the boundaries of the term “transfer” in data protection law will contribute to nuanced international data sharing rules. Indeed, in carefully narrowing the ambit of international data transfers to those uses of data that pose a prospective risk to the fundamental rights of EU data subjects, EU regulators will incentivize the design of legal and technical enclaves enabling non-EU data users to process EU data in a manner that benefits EU and non-EU communities without engendering correlative risks to individual privacy. However, ultimately, both the international community and individual countries are called upon to collaborate in raising local standards of data protection to provide minimum guarantees against State surveillance that are compatible with human and fundamental rights. At the same time, it is neither fair nor necessary to inhibit data use that enables genomic research because of incompatibilities in national legal systems protecting data subjects from surveillance and incompatibilities that arise outside the context of scientific research.

Determining the appropriate boundaries between the privacy rights of research participants and the countervailing exceptional right for State actors to access personal data that have been processed for scientific research purposes to further the interest of law enforcement raises contentious issues of public policy. A delicate balance between the public interest in scientific research and the countervailing interest in law enforcement must be achieved. Interestingly, we already see this in the context of the United States, with Certificates of Confidentiality available to protect participants from forced data disclosure by law enforcement officials [63].

Parallel progress must ideally be pursued in both of the foregoing policy arenas. That is, paths to the secure exchange of biomedical data for research purposes must be negotiated absent global consensus as to the appropriate balance between security or law enforcement interests on the one hand and data protection or privacy on the other. However, at the same time, further international dialogue must be pursued to foster an agreement on a shared minimum standard of data protection and privacy rights for individuals worldwide. To achieve this objective, it would be possible for EU regulators to issue an adequacy decision in favor of the research sector of a third country. The GDPR provides the possibility of proffering an adequacy decision in favor of only one or more specified sectors within a third country. There are good policy reasons for pursuing this path. Indeed, there have been considerable efforts on the part of scientific research communities to ensure the good governance of collaboratively generated scientific research data. International collaboration has contributed to the development of common data stewardship practices, data security standards, and biomedical research ethics rules throughout global biomedical research endeavors. It is up to lawmakers to acknowledge these efforts and bridge the gap by providing the corresponding sectoral protection of data sharing and ensuring that its processing purpose remains for scientific research in the public interest shielded from fundamental rights intrusions.

Such a development would, in the short term, constitute an appropriate recognition by lawmakers of both the positive and negative dimensions of freedom of scientific research. From a negative rights standpoint, this would protect researchers from State incursions on this fundamental right. From a positive rights standpoint, this recognition would impel scientists to pursue the dual objectives of protecting data subjects’ rights and freedoms while also excelling in the production of state-of-the-art research outputs. In the medium term, the creation of “safe data spaces” can contribute to the efficient pursuit of scientific advancement, creating a favorable regulatory environment that enables contribution to and benefit from existing scientific data resources on the part of scientific communities and the general public in compliance with clearly defined legal preconditions. In the long term, the advent of safe data spaces can significantly contribute to the formation of a novel regulatory sector in the health sciences that directs public and private resources toward judiciously balancing the interests of the main contributors and stakeholders engaged. These stakeholders include patients, research participants, researchers, and physicians. Thus, the legislator would act as a focused enabler. These developments would ultimately foster the development of a sector-specific adequacy standard in the area of health research, the foundation of which is already established in the GDPR. These considerations should serve as the beginning of a robust and global health data governance framework with standardized and binding international rules for scientific health research, including genomic science, developed and implemented in all of our interests as a global community.

## Abbreviations

CJEU: Court of Justice of the European Union
EHDS: European Health Data Space
EU: European Union
GDPR: General Data Protection Regulation
